# Two-Stage Revision Arthroplasty for Periprosthetic Hip Infection: Mean Follow-Up of Ten Years

**DOI:** 10.1155/2015/345475

**Published:** 2015-04-29

**Authors:** Szu-Yuan Chen, Chi-Chien Hu, Chun-Chieh Chen, Yu-Han Chang, Pang-Hsin Hsieh

**Affiliations:** Department of Orthopaedic Surgery, Chang Gung Memorial Hospital, Chang Gung University College of Medicine, 5 Fu-Hsing Street, Kweishan, Taoyuan 333, Taiwan

## Abstract

*Background*. Two-stage revision hip arthroplasty is the gold standard for treatment of patients with chronic periprosthetic joint infection (PJI), but few studies have reported outcomes beyond short-term follow-up. *Methods*. A total of 155 patients who underwent two-stage revision arthroplasty for chronic PJI in 157 hips were retrospectively enrolled in this study between January 2001 and December 2010. The mean patient age was 57.5 years, the mean prosthetic age was 3.6 years, and the interim interval was 17.8 weeks. These patients were followed up for an average of 9.7 years. *Results*. At the latest follow-up, 91.7% of the patients were free of infection. The mean Harris hip score improved significantly from 28.3 points before operation to 85.7 points at the latest follow-up. Radiographically, there was aseptic loosening of the stem or acetabular components in 4 patients. In the multivariate survival analysis using a Cox regression model, repeated debridement before final reconstruction, an inadequate interim period, bacteriuria or pyuria, and cirrhosis were found to be the independent risk factors for treatment failure. *Conclusion*. Our data show that two-stage revision hip arthroplasty provides reliable eradication of infection and durable reconstruction of the joint in patients with PJI caused by a variety of pathogens.

## 1. Introduction

Total hip arthroplasty is a successful procedure that provides significant pain relief and improves patients' activities of daily living. The rate of periprosthetic joint infection (PJI) after primary total hip arthroplasty (THA) has been reported to be less than 1% [1–3]. However, infection has been shown to be a devastating complication. According to the classification of Tsukayama, late chronic infection is defined as infection more than 4 weeks after the index surgery or the onset of symptoms [4]. Two-stage revision with interim antibiotic-impregnated articulating polymethyl methacrylate (PMMA) spacer implantation offers a high successful rate and is the current gold standard for treatment of a chronically infected THA.

Although this method of treatment has an infection eradication rate greater than 90% [5, 6], the outcomes beyond short-term follow-up remain largely unknown. Furthermore, treatment failure after two-stage revision is challenging to manage and is complicated by multiple morbidities because of inadequate bone stock, poor tissue integrity, surgical complexity, prolonged operation, and compromised health of patients. The purpose of this retrospective study was to report the long-term results of two-stage revision hip arthroplasty performed during a 10-year period at Chang Gung Memorial Hospital and determine the factors related to treatment failure.

## 2. Materials and Methods

PJI was diagnosed by the presence of a cutaneous sinus tract communicating with the prosthesis, isolation of the same microorganism from more than 2 cultures of intraoperative tissue specimens, purulence in the joint space, or acute inflammation defined as 5 or more polymorphonuclear leukocytes per high-power field on histopathological examination associated with abnormal erythrocyte sedimentation rate (ESR) and serum C-reactive protein (CRP) level. These diagnostic criteria were similar to the new definition of PJI proposed by Parvizi et al. [7].

The two-stage revision protocol was consistent, and all operations were performed through an anterolateral approach. The first stage consisted of a resection arthroplasty and thorough debridement followed by implantation of a temporary antibiotic-impregnated PMMA cement prosthesis. Three sets of deep tissue specimens were taken for cultures at the time of debridement. The types of antibiotic regimens were determined according to the findings on culture of specimens from the preoperative joint aspirations. If the infecting organism could not be found preoperatively, we used a combination of 4 g of vancomycin and 4 g of piperacillin per 40 g package of cement.

Custom-made silicon molds were used to form the femoral component of the cement prosthesis. The antibiotic-loaded cement was introduced into the mold in the doughy state, with a central rod pin endoskeleton placed inside, as previously detailed [8]. Fixation was achieved by manually cementing the cement prosthesis to the proximal part of the host femur. The acetabular component was made by inserting a bolus of cement into the acetabular cavity and then shaped with the use of a unipolar cup.

Intravenous antibiotics were administered for 1 week after resection arthroplasty without oral antibiotic treatment. The patients were encouraged to walk with toe-touch weight-bearing. ESR and serum CRP level were checked every 2 weeks. The second stage of the procedure was performed when the wound had healed and the ESR and serum CRP level both had returned to normal. If normalization of these infection-related parameters could not be achieved in patients with autoimmune diseases, a trend of decreased ESR and CRP level without local symptoms and signs was considered infection eradication. After second-stage reimplantation, patients were treated with prophylactic antibiotics intravenously in the immediate postoperative period.

All patients were followed up for a minimum of 3 years after the final stage of reconstruction (average, 9.7 years; range, 3 to 15 years). Institutional review board approval for the follow-up protocol and review was obtained before any patients were contacted, and written informed consent was collected from each patient before the initiation of PJI surgical treatment.

The Kaplan-Meier survival method was used to determine the cumulative probability of success. The survival end point was defined as recurrent infection when either long-term oral antibiotic suppression or repeated operations were necessary after definite implantation. Log-rank test was performed for univariate analysis, and Cox regression analysis was conducted to investigate the effects of several risk factors at the time of recurrent infection. Statistical analysis was conducted by an independent statistician blinded to surgical outcomes. *P* values < 0.05 were considered statistically significant. Statistical analysis was performed with SPSS software version 20.0 (LEAD Technologies, Inc.).

## 3. Results

A total of 155 patients (107 male and 48 female) who underwent two-stage revision arthroplasty in 157 hips (77 right side, 76 left side, and 2 bilateral) performed by a single surgeon for chronic PJI of the hips (72 primary THA, 44 revision THA, and 41 hemiarthroplasty) were included in this retrospective study from January 2001 to December 2010. The mean age of the patients was 57 years (range, 27 to 86 years), and the mean time to infection was 3.6 years (range, 1 month to 28 years). Osteonecrosis of the femur head was the most common (39%) reason for THA or hemiarthroplasty, followed by proximal femur fractures (28%), osteoarthritis (27%), ankylosing spondylitis (2%), and rheumatoid arthritis (1%). Several factors contributed to the immune-compromised status of the host, such as type 2 diabetes mellitus (24%), asymptomatic bacteriuria or pyuria (19%), cirrhosis (17%), and autoimmune diseases (5%; 3 cases of rheumatoid arthritis, 2 cases of ankylosing spondylitis, 2 cases of systemic lupus erythematosus, and 1 case of psoriasis), and 3 patients had end-stage renal disease requiring maintenance hemodialysis (Table 1).

The temporary cement spacers were retained for an average of 18 weeks (range, 4 weeks to 3 years). After resection arthroplasty, 5.7% of patients presented with recurrent infection and needed repeated debridement (2 patients) or exchange cement prosthesis (7 patients) before reimplantation. During final reconstruction, revision arthroplasty could be fixed with the press-fit technique in 78% of patients due to little acetabular and femoral bone loss. Hybrid cementation and fully cemented technique were applied in 20% and 2% of patients, respectively. Bone grafting during reconstruction was required in 20% of patients, and a morselized allograft could achieve this need because of minimal acetabular bone defects in most circumstances; only 2% of patients needed a structural allograft for Paprosky et al. [9] 2B or C acetabular bone defects. Acetabular antiprotrusio devices were needed in 13% of patients for Paprosky 3A or B massive acetabular bone loss. An allograft-prosthesis composite and megaprosthesis were used in 4% of patients for Paprosky 3B or type 4 femoral defects after thorough debridement and removal of PMMA cement spacers (Table 2).

A total of 71% of PJIs were microbiologically confirmed; coagulase-negative staphylococci were the most common pathogen (22%), followed by* Staphylococcus aureus* (21%), gram-negative bacilli (13%), and anaerobes (7%). Although 3 sets of deep tissue specimens were taken routinely for cultures at the time of debridement, 29% failed to show growth on aerobic, anaerobic, mycobacterial, and fungal cultures submitted to the microbiology laboratory; culture-negative (CN) PJI was thus diagnosed. Microbiologic laboratory data are summarized in Table 3.

**Figure 1:**
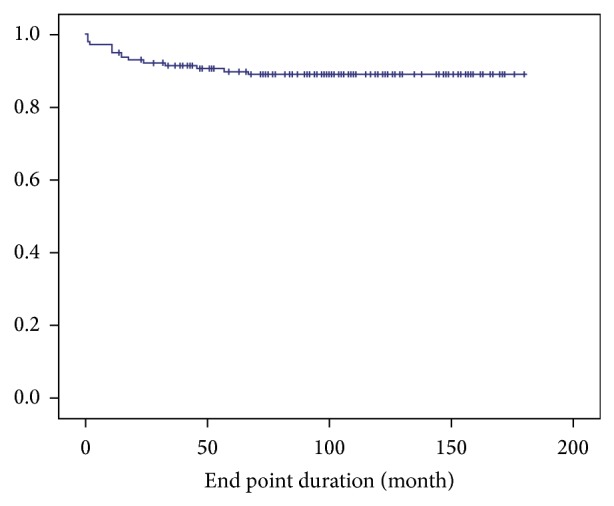
Kaplan-Meier curve for recurrent infection-free survival. In total, 91.7% of 155 patients with periprosthetic joint infection remained clinically free of infection over the entire follow-up period. The survivorship without recurrent infection was 96.8% after 1 year and 94.3% after 3 years and became stationary at 91.7% after 5.5 years.

After an average of 9.7 years of follow-up, 91.7% of the 155 patients with PJI remained clinically free of infection. The Kaplan-Meier curve for recurrent infection-free survival is shown in Figure 1. The probability of survival without recurrent infection was 96.8% after 1 year and 94.3% after 3 years and became stationary at 91.7% after 5.5 years. The average interval between final reimplantation and recurrent infection was 26.2 months (range, 1 to 67 months), and 1 patient developed a deep infection complicated by sepsis and mortality (0.6% disease-specific mortality). Postoperatively, 1 patient had a periprosthetic fracture due to a ground-level fall accident, 3 patients had recurrent dislocation, and 4 patients had aseptic loosening of the stem or acetabular component; the complication rate was 5%. Another 24 patients died at an average of 72 months after final reconstruction for various reasons (14 natural deaths, 4 cases of acute myocardial infarction, 2 cases of hepatocellular carcinoma, 1 case of rectal adenocarcinoma, 1 case of acute myeloid leukemia, 1 case of meningitis, and 1 case of perforated peptic ulcer). No patients were lost to follow-up, and the overall mortality rate was 16.1%. The mean Harris hip score improved from 28.3 points (0 to 39) before operation to 85.7 points (47 to 100) at the latest follow-up. Statistical analysis showed a significant improvement (paired-samples *t*-test, *P* < 0.001).

Table 4 presents Kaplan-Meier analyses with log-rank test for treatment failure. There were several noncontributing variables that were not predictive of survival, including toxicity of different pathogens (inclusive of methicillin-resistant* Staphylococcus aureus*, gram-negative bacilli, anaerobes, or CN PJI), patient age, laterality, body mass index, cigarette smoking, diabetes mellitus, autoimmune diseases, end-stage renal disease, underlying malignancy, cementation, bone grafting, and ESR or CRP level before final reimplantation. The univariate risk factor analyses showed a significantly lower survival rate in male patients (10-year infection-free survival rate, 86%; *P* = 0.037), an inadequate interim period (less than 3 months) (10-year infection-free survival rate, 85%; *P* = 0.036), repeated debridement before final reconstruction (10-year infection-free survival rate, 73%; *P* = 0.047), cirrhosis (10-year infection-free survival rate, 71%; *P* = 0.001), or bacteriuria or pyuria at reimplantation (10-year infection-free survival rate, 67%; *P* = 0.015).

In the multivariate survival analysis using the Cox regression model, repeated debridement before final reconstruction (hazard ratio, 10.6; 95% confidence interval, 2.4 to 46.2; *P* = 0.002), an inadequate interim period (hazard ratio, 5.9; 95% confidence interval, 1.4 to 24.0; *P* = 0.014), bacteriuria or pyuria (hazard ratio, 5.6; 95% confidence interval, 1.4 to 23.0; *P* = 0.017), and cirrhosis (hazard ratio, 3.1; 95% confidence interval, 1.1 to 9.0; *P* = 0.033) were independent risk factors for treatment failure (Table 5).

## 4. Discussion

To use an articulating, antibiotic-loaded PMMA cement spacer is a simple and fast molding method to fit all defects and allows early mobilization and efficient local antibiotic delivery [10–14]. The two-stage revision hip arthroplasty protocol offers the greatest chance for eradication of infection, with reported success rates of greater than 90% in various studies [5, 6]. Although it has been the gold standard for the treatment of patients with chronic PJI, few studies have reported outcomes beyond short-term follow-up.

In 1989, Wilson and Dorr [15] followed up 22 patients for a minimum of 3 years and found a recurrent infection rate of 9%. Nestor et al. [16] followed up 34 patients and reported a high recurrent infection rate of 18% in 1994. Lai et al. [17] documented an infection recurrence rate of 12.5% among 40 patients followed up for an average of 4 years (range, 2.5 to 7 years) in 1996. Haddad et al. [18] similarly followed up 50 patients for an average of 5.8 years (range, 2 to 8.7 years) and found an infection recurrence rate of 8% in 2000. Durbhakula et al. [19] reported a 100% success rate in 20 patients followed up for an average of 38 months (range, 26 to 67 months) in 2004. In the next year, Hofmann et al. [20] showed that 94% of patients with PJI remained clinically free of infection at an average of 76 months (range, 28 to 148 months) postoperatively. Masri et al. [21] followed up 29 patients for an average of 47 months (range, 24 to 88 months) and found an infection recurrence rate of 10.3% in 2007.

Because of sample size constraints, the authors were unable to perform multivariate analyses to identify independent risk factors for treatment failure in these studies [15–21]. To the best of our knowledge, our study includes the most cases (155 patients) with the longest duration of follow-up (average, 9.7 years; range, 3 to 15 years) treated by a single surgeon with a consistent treatment protocol. The results of this study showed that 91.7% of 155 patients with PJI remain clinically free of infection after mean follow-up of ten years, and the study had enough power to determine factors contributing to treatment failure. A significantly lower survival rate was identified in patients with repeated debridement before final reconstruction, an inadequate interim period (less than 3 months), bacteriuria or pyuria at reimplantation, or cirrhosis.

There is little evidence in the literature regarding a definite interim period between resection arthroplasty and reimplantation in a two-stage protocol. Lai et al. [17] performed revision THA at an average interval of 48 (range, 8 to 108) weeks. Haddad et al. [18] used an interim period of 3 weeks, which was delayed in some patients with poor wound healing or a medical comorbidity. The planned interval in the study by Fisman et al. [22] was 2 months. The average interval between stages was 12.5 weeks (range, 10 to 21 weeks) in the protocol of Durbhakula et al. [19] in 2004. Hofmann et al. [20] implanted prosthesis at an average of 14 weeks (range, 3 to 49 weeks). Cordero-Ampuero et al. [23] delayed reimplantation surgery until clinical and serological normalization had been achieved, so the mean interval was 10 months (range, 2 to 24 months). Masri et al. [21] had an average interim period of 5.5 months (range, 2.5 to 20 months). The average time from prosthesis removal to reimplantation was 3.4 months (range, 1.1 to 16.3 months) in the study by Toulson et al. [24]. The average interval in our study was 18 weeks, with a wide range of 4 weeks to 3 years. We analyzed different interim periods inclusive of 42 days, 60 days, 90 days, and 120 days and found that an inadequate interim period (less than 90 days) is a major independent risk factor for treatment failure (hazard ratio, 5.9; 95% confidence interval, 1.4 to 24.0; *P* = 0.014). Abrupt reimplantation adversely affects treatment outcomes because of an insufficient therapeutic requirement for local antibiotics released from the PMMA cement spacer and an inadequate duration of antimicrobial activity beyond the initial period of spacer implantation [25]. Our data indicate that interim period in a two-stage revision protocol should exceed 3 months.

A number of studies have evaluated the relationship between urinary tract infection and PJI, but there has been little focus on bacteriuria or pyuria and two-stage revision. In regard to the pathophysiology between PJI and urinary tract infection, patients with bacteriuria are likely to have bacteremia through traumatized genitourinary mucosa, invading the systemic circulation and becoming the infection foci at remote anatomic locations [26]. Asymptomatic bacteriuria is possibly a reservoir for bacterial contamination of the wound or may simply identify a patient at increased risk for infection at any site [27, 28]. Because bacteriuria or pyuria in many patients is dynamic, with spontaneous resolution or reinfection [29], we chose microscopic urinalysis and leukocyte esterase dipstick tests for screening before definite implantation. This study shows that bacteriuria or pyuria is another important risk factor for recurrent infection after two-stage revision (10-year recurrent infection-free survival rate, 67%; *P* = 0.015) and delayed reimplantation until eradication of bacteriuria is recommended.

Cirrhosis is a well-known factor contributing to an immunocompromised state because of increased shunting of blood away from the liver, impairment of neutrophil function, a decrease in the removal capacity of the reticuloendothelial system, decreased opsonization capacity, and increased intestinal permeability of bacteria and associated endotoxins [30]. It is also an identifiable factor contributing to periprosthetic infection [31]. Our data indicate a significantly lower 10-year recurrent infection-free survival rate (80%, *P* = 0.001) in 26 patients with cirrhosis (12 with Child A, 9 with Child B, and 5 with Child C).

Devitalized tissue and biofilm cannot be thoroughly removed by arthrotomy and debridement alone. Radical debridement and implant removal are mandatory for infection-free survival, and the quality of debridement is likely to affect the outcome. Although it is difficult to evaluate directly, we used the need for repetitive operations as a parameter to assess the quality of debridement. This study showed that repeated debridement or spacer exchange before final reconstruction is a risk factor for treatment failure (10-year recurrent infection-free survival rate, 72.7%; *P* = 0.047). This explains that the need for repeated surgeries implies poor quality of debridement, and the probability of recurrent infection is significantly elevated. Based on this observation, it seems reasonable that various pathogens (methicillin-resistant* Staphylococcus aureus*, gram-negative bacilli, anaerobes, or CN PJI) are not predictive of survival if radical debridement can be achieved. This finding is consistent with previous studies that gram-negative bacilli [32, 33] and CN PJI [34] are associated with a favorable outcome after two-stage revision which are comparable to that associated with PJI due to gram-positive and known pathogens, respectively. The 10-year infection-free survival rate is slightly lower in patients with methicillin-resistant* Staphylococcus aureus* PJI (80%); however, significant difference is not achieved (*P* = 0.107).

This study had some limitations. First, the retrospective study design allowed potential selection biases, although we tried to minimize biases with practice by a single surgeon. Second, because of the acceptable functional results after articulating PMMA cement spacer implantation in some patients, it is difficult to standardize the interval between stages. The mean duration between stages was 18 weeks (range, 4 weeks to 3 years). Third, the percentage of CN PJI in our study was high (29%), and we should use every possible strategy to improve our culture yield rate in the future, inclusive of extracted implant sonication [32], polymerase chain reaction assay of periprosthetic tissue and synovial fluid for every case of CN PJI, an extended period of culture incubation for at least 14 days and possibly 21 days, and withholding antimicrobial therapy for at least 2 weeks before the collection of periprosthetic tissue or synovial fluid.

Several major questions regarding two-stage revision remain unanswered, such as the optimal duration of intravenous antibiotic use and whether oral antibiotics should be used during the interim period and after final reconstruction. Since 1996, we have used intravenous antibiotics for only 1 week after resection arthroplasty and in the immediate postoperative period after the second stage of revision. This has provided consistently optimal treatment of infection over the ten-year follow-up period, and the importance of use of systemic antibiotics seemed to decrease after the use of an antibiotic-impregnated PMMA cement spacer. We believe that the long-term success of a two-stage revision hip arthroplasty protocol is achieved by radical debridement, local release of a high concentration of antibiotics from the PMMA cement spacer, an adjustable regimen of antibiotics in cement, an easy spacer exchange, and easy reimplantation of the prosthesis on final reconstruction. In the literature, there has also been increasing evidence for one-stage revision arthroplasty to treat PJI in selected patients with infection-free survival rates ranging from 83% to 100% [35–37].

This study includes the largest number of cases and the longest duration of follow-up reported to date and was designed to identify variables predictive of infection-free survival in the evaluation of two-stage revision hip arthroplasty for patients with chronic PJI. The results showed several variables on univariate analysis that predict survival: male gender, an inadequate interim period (less than 3 months), repeated debridement, cirrhosis, and bacteriuria or pyuria at reimplantation. Multivariate analyses showed that repeated debridement, an inadequate interim period (less than 3 months), cirrhosis, and bacteriuria or pyuria at reimplantation are the most important risk factors for recurrent infection after two-stage revision protocol. In conclusion, our data show that two-stage revision hip arthroplasty provides reliable eradication of infection and durable reconstruction of the joint, and it remains the treatment of choice in patients with chronic hip PJI caused by a variety of pathogens.

## Figures and Tables

**Table 1:** Patient characteristics.

Age (years)	57.5 (27–86)
Male gender	107 (69%)
Body mass index (kg/m^2^)	24.5 ± 4.3 (14.2–37.3)
Cigarette smoking (>1 pack per day)	69 (45%)
Laterality (right/left/bilateral)	77 (49.7%)/76 (48.4%)/2 (1.3%)
Diabetes mellitus	37 (23.8%)
End-stage renal disease	3 (1.9%)
Autoimmune diseases	8 (5.2%)
Cirrhosis (Child A/Child B/Child C)	12 (7.7%)/9 (5.8%)/5 (3.2%)
Bacteriuria or pyuria at reimplantation	29 (18.5%)
Underlying malignancy	4 (2.6%)
Prosthesis age (years)	3.6 (30 days to 28 years)
Interim period (weeks)	17.8 (4 weeks to 3.2 years)
Follow-up duration (years)	9.7 (3–15)

Data are mean (range), mean ± standard deviation (range), or number (%) of episodes.

**Table 2:** Diagnosis and surgical procedures.

Diseases for arthroplasty (osteonecrosis/fracture/osteoarthritis/autoimmune/other)	62 (39%)/44 (28%)/43 (27%)/5 (3%)/3 (2%)
Index procedure (primary THA/revised THA/hemiarthroplasty)	72 (46%)/44 (28%)/41 (26%)
Cementation in index OP (cementless/hybrid/full cementation)	129 (82%)/22 (14%)/6 (4%)
C-reactive protein level before revision OP (mg/L)	10.5 ± 17 (0.3–96)
Erythrocyte sedimentation rate before revision OP (mm/hr)	25.1 ± 18 (2–72)
Revision prosthesis (cage/megaprosthesis)	21 (13%)/1 (0.6%)
Cementation in revision (cementless/hybrid/full cementation)	122 (78%)/31 (20%)/4 (2%)
Bone grafting in revision (no/morselized allograft/onlay allograft/allograft-prosthesis composite)	125 (79%)/26 (17%)/3 (2%)/3 (2%)

Data are number (%) of episodes or mean ± standard deviation (range).

THA: total hip arthroplasty; OP: operation.

**Table 3:** Microbiologic laboratory data.

Pathogens	Number (%) of episodes
Methicillin-sensitive *Staphylococcus aureus *	19 (11.5%)
Methicillin-resistant* Staphylococcus aureus *	15 (9.1%)
Coagulase-negative staphylococci	36 (21.9%)

Gram-negative bacilli	
*Escherichia coli *	7 (4.2%)
*Pseudomonas aeruginosa *	4 (2.4%)
*Klebsiella pneumoniae *	3 (1.8%)
*Salmonella *	2 (1.2%)
*Serratia marcescens *	2 (1.2%)
*Morganella morganii *	1 (0.6%)
*Aeromonas hydrophila *	1 (0.6%)
*Acinetobacter baumannii *	1 (0.6%)

Anaerobes	
*Peptostreptococcus *	4 (2.4%)
*Enterococcus faecalis *	4 (2.4%)
*Propionibacterium acnes *	3 (1.8%)

*β*-streptococcus group B	3 (1.8%)
*Viridans streptococcus *	3 (1.8%)
*Streptococcus pneumoniae *	1 (0.6%)
*Staphylococcus epidermidis *	1 (0.6%)
*Mycobacterium tuberculosis* complex	1 (0.6%)
No growth	46 (29.3%)

**Table 4:** Noncontributing variables.

Variables	*P* value
Age (<60/>60 years)	0.575
Body mass index (<30/>30 kg/m^2^)	0.837
Cigarette smoking (<1/≥1 pack per day)	0.171
Laterality	0.159
Diabetes mellitus	0.082
End-stage renal disease	0.571
Autoimmune diseases	0.495
Diagnosis for arthroplasty	0.640
Primary prosthesis	0.460
Cementation in primary operation	0.598
Methicillin-resistant *Staphylococcus aureus* as pathogen	0.107
Anaerobes as pathogen	0.275
Gram-negative bacillus as pathogen	0.200
Culture-negative periprosthetic joint infection	0.074
C-reactive protein level before revision (<10/>10 mg/L)^#^	0.894
Erythrocyte sedimentation rate before revision (<40/>40 mm/hr)^∧^	0.535
Revision prosthesis	0.571
Cementation in revision	0.541
Bone grafting in revision	0.511

Log-rank test for Kaplan-Meier survivorship; a *P* value of <0.05 was considered to be statistically significant.

^#^The cutoff value is 5 mg/L.

^∧^The cutoff value is 20 mm/hr.

**Table 5:** Multivariate survival analysis.

Variables	Hazard ratio	95% confidence interval	*P* value
Repeated debridement before final reconstruction	10.6	2.4–46.2	0.002^*^
Interim period less than 3 months	5.9	1.4–24.0	0.014^*^
Bacteriuria or pyuria	5.6	1.4–23.0	0.017^*^
Cirrhosis	3.1	1.1–9.0	0.033^*^
Male gender	3.9	0.5–31.1	0.200

Cox regression model.

^*^A *P* value of <0.05 was considered to be statistically significant.
